# Water-Processable Intercalated Graphitic Pyroproteins and Electromechanic Properties for Smart Structural Bricks

**DOI:** 10.1021/acsomega.5c07891

**Published:** 2025-10-27

**Authors:** Maria Caporali, Daniel A. Triana-Camacho, Rocco Malaspina, Antonella D’Alessandro, Andrea Meoni, Andrea Ienco, Stefano Martinuzzi, Martina Banchelli, Filippo Ubertini, Luca Valentini

**Affiliations:** a Institute of Chemistry of OrganoMetallic Compounds-ICCOM, 9327 National Research Council-CNR, Sesto Fiorentino 50019, Italy; b Department of Civil and Environmental Engineering, 9309 University of Perugia, Via G. Duranti, Perugia 06125, Italy; c Department of Physics and Geology, 9309 University of Perugia, Via A. Pascoli, Perugia 06123, Italy; d Institute of Applied Physics Nello Carrara-IFAC, 9327 National Research Council-CNR, Via Madonna del Piano 10, Sesto Fiorentino 50019, Italy

## Abstract

This study introduces a novel method for synthesizing phosphate-intercalated graphitic pyroprotein (P-GPy) using the regeneration process of silk fibroin (SF). SF was prepared by mixing degummed silk fibers and calcium chloride (CaCl_2_) in formic acid, resulting in a silk I-like conformation, which was then converted into silk II by being redissolved in phosphate buffer (PBS). We find that the β-sheet structure of silk II is transformed to an sp^2^-hybridized carbon structure by simple heating. Techniques such as Raman, XRD, and SEM with EDS mapping demonstrate successful creation of P-GPy characterized by a uniform phosphate distribution. We demonstrate the exfoliation in water of P-GPy in layered graphene sheets. SF dissolved in PBS was added to clay, and the thermal treatment required for firing the refractory bricks is used to obtain P-GPy. Electrical impedance spectroscopy was employed to investigate the piezo-impedance and piezoresistive behaviors of clay-based P-GPy composites, demonstrating the potential of the fabricated bricks to function as smart strain sensors for structural health monitoring.

## Graphical abstract

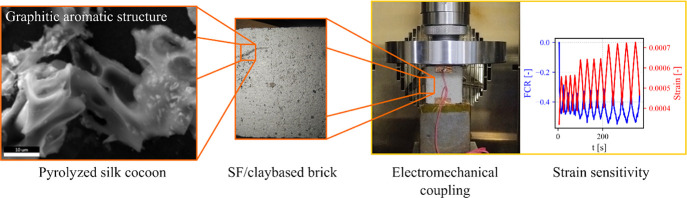

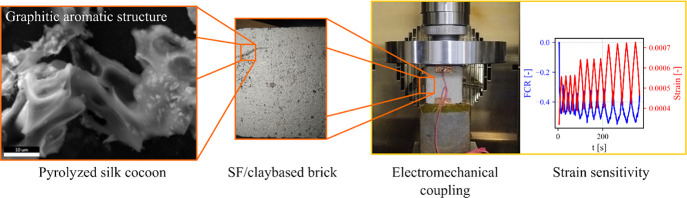

## 1 Introduction

Silk fibroin is a biological macromolecule linked by peptide bonds of various amino acids with their own characteristic composition, which results in the arrangement of an amino sequence and the formation of the distinctive secondary structures, such as α-helices, β-sheets, and turns (or loops).^1–3^

A recent study reported the discovery of a new 2D crystalline phase of self-assembled protein on vdW solids with an epitaxial relationship to the underlying lattice.^4^ From a protein solution, the new 2D nanostructures were obtained through surface-directed assembly and folding of the molecules with a final architecture composed of fully ordered monolayers of β-sheet lamellae. The carbonization of β-sheet-rich proteins has been demonstrated as a method to transform the C–C bonds into aromatic molecules, which may stack to form pseudo graphitic materials.^5^ Moreover, it was observed that some β-sheet-rich protein *Bombyx mori* silk fibers after thermal treatment are fully pyrolyzed into their carbonaceous forms.^6^ In a recent study, it was demonstrated that the silk I structure of SF is the key secondary structure that promotes the dissolution of SF films in PBS for the regeneration of water-stable silk biomaterials with moderate β-sheet content.^7^

The silk structural features composed of hard β-sheet crystals as well as their heat transformation open up the possibility of the use of bioderived materials to mimic the structure of two-dimensional (2D) nanomaterials.

The intercalation of atoms between the interfaces of 2D layered graphene has mainly focused on alkali metals and transition atoms.^8–13^ However, the intercalation of molecules through layered graphene remains challenging;^14–24^ this is because the size and energy barrier for intercalation inhibit the permeation of such large molecules.^25^

The confinement of molecules through liquid solutions of SF is a valid pathway to circumvent the above limitations. That is, the molecules would first intercalate the chemical species that recombine at the interface via thermal treatment.

Taking advantage of fibroin’s transformation into graphitic structures, and considering that clay’s transformation also requires thermal treatment, the combination of these materials can lead to electrically sensitive bricks with potential applications in structural health monitoring (SHM) of brick masonry constructions. This intriguing area of research has been studied in a few papers addressing the combination of clay- and carbon-based composites. For instance, Tang et al. demonstrated that firing graphene oxide (GO)/clay-based bricks in a controlled atmosphere improves electrical resistivity for specimens with more than 0.5 wt % of GO.^26^ Along the same lines, reduced GO (rGO) has been incorporated into clay-based bricks, as its carboxylate groups promote interactions with clay-H_2_O products,^27^ consequently enhancing physicochemical properties of these clay-based composites.

In this work, we report a mechanism of liquid phase intercalation of PBS to form confined phosphate ions at the interface of graphitic pyroproteins (P-GPy) with a route that exploits the initial state of the large intercalant molecule (e.g., PBS) itself. It was then demonstrated that P-GPy can be exfoliated in water.

Finally, such processable water silk fibroin can be processed with clays, which are traditionally used in the construction industry, to obtain fired structural bricks containing P-GPy demonstrating the potential of such fired bricks to reliably function as strain sensors for structural health monitoring in masonry constructions. This approach paves the way for an alternative, more effective, and eco-friendly design of smart bricks that avoids the use of metallic and poorly dispersible microfibers, as adopted in previous studies by the authors.^28^

## 2 Experimental Details

### 2.1 Materials

*Bombyx mori* silk cocoons were supplied by a local farm (Fimo Srl, Milano, Italy), while calcium chloride (CaCl_2_), formic acid (FA), and sodium bicarbonate (NaHCO_3_) were supplied by Sigma-Aldrich. The cocoons were degummed in boiling aqueous NaHCO_3_ solution (5 g in 200 mL of water) for 30 min. After rinsing, 0.70 g of silk fibers was dissolved in 5 mL of FA by adding CaCl_2_ in a weight ratio of 70/30 with respect to the silk amount (the silk fibroin concentration is 140 mg/mL).^29^ The SF solution was then sonicated in an ultrasonic bath for 2 h. SF films were produced by leaving the SF solutions to evaporate onto Petri dishes for 48 h to remove any trace of the solvent (Figure S1). SF films were then redissolved in PBS 1× (pH 7.4) to get the same concentration of the FA solution and sonicated for 2 h at room temperature in an ultrasonic bath.

### 2.2 Pyroprotein for Bricks

The pyroprotein samples were prepared using raw silk cocoons and regenerated silk fibroin from the PBS solution. The latter was first transferred in an alumina crucible and heated at *T* = 35 °C for 24 h until a dried white solid was obtained. Approximately 20 mg of silk cocoon and the regenerated silk was placed in two separate alumina crucibles. The samples were heated to the desired temperature, *T* = 600 °C, at a rate of 10 °C min^–1^ for 2 h in a nitrogen atmosphere. Piezoresistive clay bricks with dimensions of 5 cm × 5 cm × 7 cm were fabricated following the elaboration process reported by Meoni et al.^30^ However, in this study, bricks were prepared by manually mixing fresh clay with the SF in PBS solution concentrated at 0.1 wt %. The bricks were then dried at 90 °C for 6 h and subsequently fired at 900 °C for an additional 6 h. The last heat treatment was conducted by immersing the brick samples in refractory crucibles filled with carbon coke powder. This allowed the brick samples to be fired under a reduced atmosphere. Copper tape electrodes, 0.05 mm thick, were adhered to the opposite largest faces of the bricks when cooled. Therefore, three plain bricks were prepared and denoted as plain-coke bricks (PCB), while the three SF-coke bricks were labeled as SFCB. For simplicity, the authors refer to these specimens collectively as “smart bricks”.

### 2.3 Liquid Phase Exfoliation of the Pyroprotein Derived from Regenerated Silk Fibroin

The pyroprotein obtained from calcination was washed with deionized water (10 mL, four times) and finally with acetone and then dried overnight by vacuum pump. Then, 5 mg of the isolated pyroprotein was suspended in 5 mL of MQ water and kept under the action of ultrasounds for 5 h, keeping the temperature of the ultrasonic bath constant at 20 °C. Afterward, 5 mL of water was added to the suspension and by centrifugation a sediment was obtained that was washed further with acetone (10 mL) and the final black residue was dried under air. To improve further the exfoliation degree, the suspension of the pyroprotein in water was kept under the action of ultrasounds for a total of 72 h at 20 °C, and as a comparison, a suspension of the pyroprotein in dimethyl sulfoxide having the same concentration (1 mg/mL) was also subjected to ultrasounds for 72 h at 20 °C. Afterward, the sample was washed once with water, once with ethanol, and finally dried under vacuum.

### 2.4 Material Characterizations

X-ray diffraction spectra were collected on the powder samples with an X’Pert PRO diffractometer, operating in Bragg–Brentano parafocusing geometry with a Cu target and Kα radiation (λ = 1.5418) at 40 kV beam voltage and 40 mA current. The data were collected in the 5–90° 2θ range, with steps of 0.01° and a counting time of 130 s.

Scanning electron microscopy and EDX experiments on pyrolyzed silk cocoon and pyroproteins were carried out using a dual-beam TESCAN GAIA3 FIB/SEM ultrahigh-resolution field emission microscope at 5 keV on the powder samples.

Raman measurements of the samples were performed at room temperature in a backscattering configuration using a LabRAM HR 800EVO Raman spectrometer (Horiba France SAS) equipped with an Olympus BXFM microscope (objective X100, NA 0.9), a TE-cooled CCD detector (Syncerity OE), a 633 nm HeNe laser, and 600 grooves/mm diffraction grating. The spectral resolution was 0.9–1.8 cm^–1^. The samples suspended in methanol were drop-cast on silicon wafers as the substrate and then dried under a stream of nitrogen. The laser power at the sample was 0.7 mW, and the acquisition time was 1 s. Ten to 20 spectra were registered for each sample at different locations to verify sample homogeneity and the absence of photoinduced phenomena. The reference spectrum of Si was measured contextually in each sample. Raman spectra were corrected for the baseline, and peak analysis was performed through the Gaussian fitting function to calculate the position, intensity, and area of the characteristic peaks. For all investigated samples, four Gaussian-shaped bands G, D1, D2, D3, and D4 were used for the spectral analysis where G, D1, D2, D3, and D4 are attributed to the ideal graphitic lattice, disordered graphitic lattice,^31^ disordered graphitic lattice (surface graphene layers),^31^ amorphous carbon,^32^ and disordered graphitic lattice (A1g symmetry),^33,34^ respectively.

Infrared spectra were recorded by using a Fourier transform spectrometer from Jasco equipped with its diamond ATR (attenuated total reflection) module. The spectra were recorded in the 4000–400 cm^–1^ spectral range at a resolution of 2 cm^–1^. Each measured spectrum was averaged from 300 scans. A background spectrum without a sample was acquired using the same number of scans before each measurement. Spectral data were preprocessed by tracing a straight baseline from 1740 to 1560 cm^–1^. To estimate the different components of the amide I spectral profiles, a curve-fitting procedure was employed. Each component was assigned a Gaussian line shape, a full width at half height (fwhh) fixed at 20 cm^–1^, and the weight was determined without constrains.

### 2.5 Mechanical Measurements on Bricks

Smart bricks were tested using an Instron 68TM-50 universal testing machine (UTM) (Figure 1a). The specimens were characterized by using two different mechanical profiles. First, specimens were subjected to step loading, with each load maintained for 4 min. This type of measurement was used in parallel with the Electro Impedance Spectroscopy (EIS) technique. The characterization began with a preload of 0.025 kN, followed by incremental loads of 1, 2, 3, 4, and 5 kN (Figure 1b). The UTM was equipped with an Instron thermal chamber (model 3119-600), which was used to control the temperature of the smart bricks during both mechanical and electrical testing (described later below). The investigated temperature was 25 °C. In addition, the temperature was controlled using Bluehill software. Then, a Tinytag logger was placed inside the chamber to monitor both the temperature and humidity. To ensure thermal equilibrium between the chamber and the samples, the smart bricks were left inside the thermal chamber for 10 h before each test. This procedure allowed mechanical and electrical measurements to be conducted under stable thermal conditions the next day.

**Figure 1:**
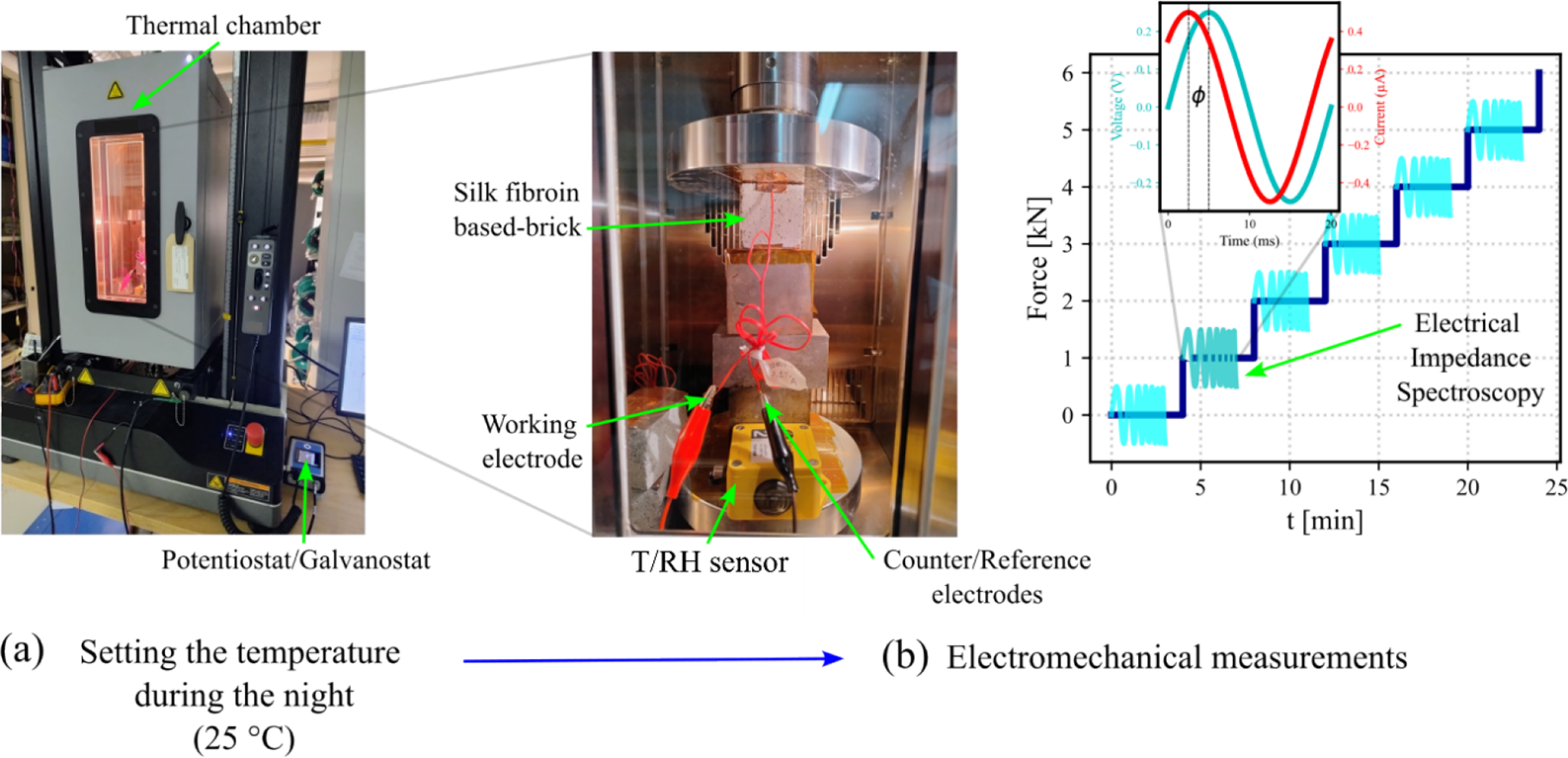
Experimental setup for combined electrical impedance spectroscopy (EIS) and mechanical measurements at 25 °C. (a) Temperature conditioning phase (10 h). (b) Mechanical stair-step loading profile from 0.02 to 5 kN, with EIS performed during each step for 4 min.

The second mechanical profile consisted of a preload ramp from 0.02 to 1 kN, followed by a series of four loading cycles ranging from 1 to 3 kN, and then from 1 to 4 kN, and finally from 1 to 5 kN. All the progressively increasing cyclic loading profile maintained a force-controlled rate of 200 N/s.^28^

### 2.6 Electrical Measurements on Bricks

EIS measurements were conducted using a PalmSens4 Potentiostat/Galvanostat simultaneously with step loading. The frequency range extended from 0.1 Hz to 1 MHz, with a total of 71 data points acquired, corresponding to 10 points per decade. A sinusoidal excitation signal with an amplitude of 250 mV was applied. The reference and counter electrodes were combined and connected to the top electrode of the smart brick, while the working electrode was connected directly to the bottom electrode, as shown in Figure 1b. Additionally, the ground was connected to the chamber, which was used as a Faraday cage to minimize any external electromagnetic interference.

Data measured by EIS at different forces and strains were optimized using the equivalent circuit models shown in Figure 2. The software tool used for such an optimization was the Equivalent Circuit Analysis tool in PSTrace5.11 by PalmSens. The set {R1, Q1, and R3} forms a Cole–Cole element, which depicts the dielectric behavior of clay-based bricks at high frequency, with R1 and R3 being the electrical resistances and Q1 the pseudoadmittance at high frequency. The circuits in Figure 2a and Figure 2b were used to optimize impedance curves of PCB and SFCB, respectively. However, the circuit in Figure 2b can be considered as the general model, whereas Figure 2a represents a simplification in which the constant phase element (CPE) associated with Q3 is replaced by a resistor of 0 Ω. This pseudoadmittance Q3 was incorporated into the model because the impedance curves of SFCB presented a diffusion element at the low-frequency range.

**Figure 2:**
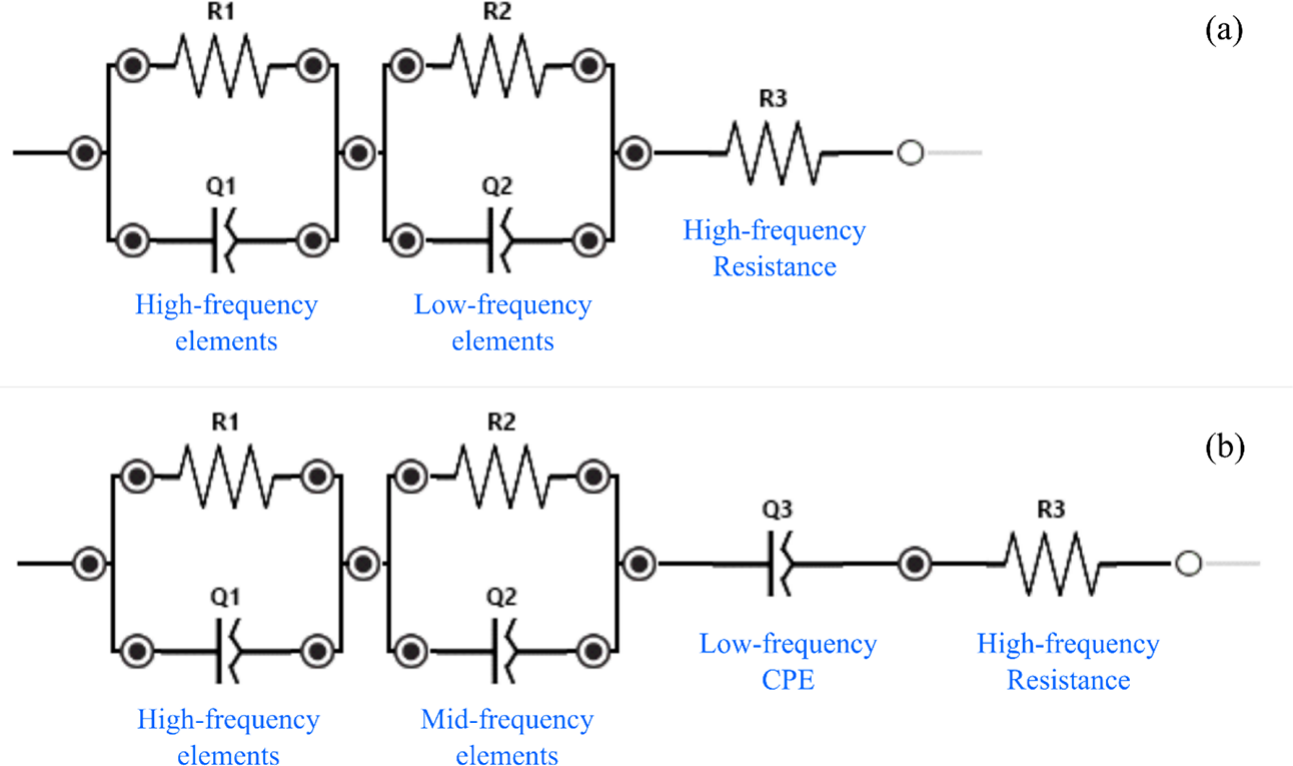
Circuit models implemented to describe the impedance spectra of (a) PCB and (b) SFCB.

A biphasic approach previously used to characterize the piezoresistance of silk fibroin/mortars^35^ was here implemented in combination with the progressively increasing cyclic loading profile. In this context, a signal generator provided a ±10 V signal square wave at 1 Hz directly on a circuit formed by the silk fibroin/brick in series with a conventional gain resistor (*R*_G_) of 20 MΩ. Then, the NI-PXIe (1092) acquisition system was used to acquire electrical voltage from both the gain resistor (*V*_CH1_) and smart brick (*V*_CH0_). By means of Ohm’s law, the electrical current flowing across the circuit was calculated, as described in eq 1:

$$i=\frac{V_{\mathrm{CH}1}}{R_{G}}$$ 1

Consequently, electrical resistance of the smart brick (*R*) was calculated using Ohm’s law, as presented in eq 2

$$R=\frac{V_{\mathrm{CH}0}}{i}$$ 2

The electrical resistance of smart bricks was finally sampled every second during the steady-state phase of the electrical current.

## 3 Results

### 3.1 Characterization of Heat-Treated Silkworm Cocoon

Previously, silk cocoons were used for synthesizing graphene;^36–39^ the process is basically a process of biocharing where the silk cocoon is pyrolyzed in an inert atmosphere to get graphene.

The microstructure and composition of the pyrolyzed silk cocoon was analyzed through SEM and EDX analyses, shown in Figure 3a–d and Table 1. Bubble-like structures form, introducing microscale pores with fiberlike morphologies surrounding central voids reminiscent of the fibers of the cocoon (Figure S1). Bright regions visible in the SEM image are likely caused by electron beam charging.^38^

**Figure 3:**
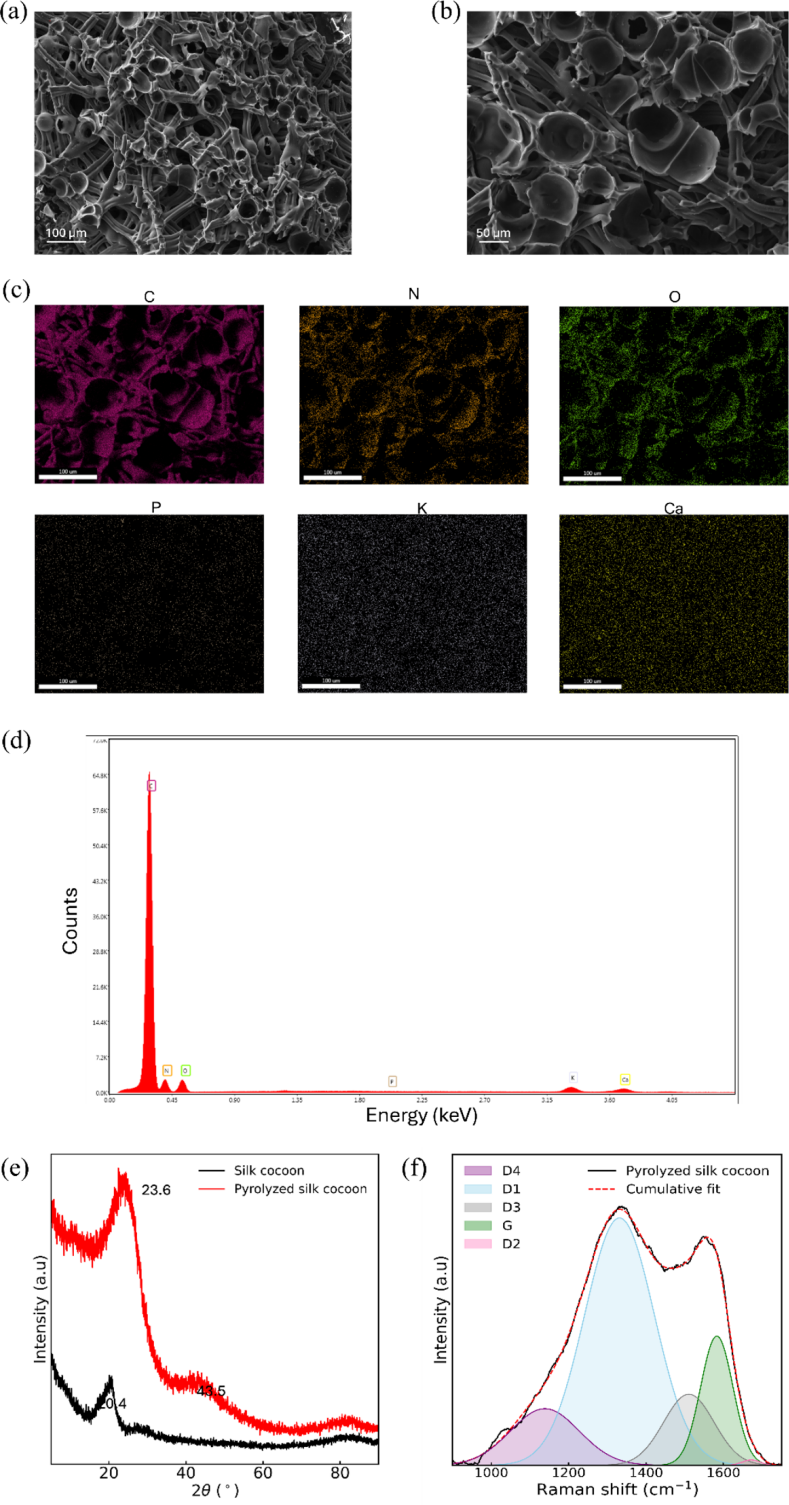
(a, b) SEM images of pyrolyzed silk cocoon. (c) EDX mapping and (d) spectrum of pyrolyzed cocoon, (e) X-ray diffraction profiles of pristine and pyrolyzed silk cocoon, and (f) deconvoluted Raman spectra of pyrolyzed silk cocoon excited at 633 nm.

**Table 1:** EDX Elemental Analysis of a Pyrolyzed Cocoon

element	weight %	atomic %
C	68.74	74.31
Ca	1.74	0.56
K	1.91	0.63
N	18.16	16.83
O	9.44	7.66
P	0.01	0.00

An XRD pattern of pyrolyzed silk cocoon shows two peaks at around 2θ = 24 and 43° (Figure 3e). The peak at 24° results from the growth of the crystalline carbon domain.^40^ However, the appearance of the peak at 2θ = 43° suggests that all six-member carbon atoms in the aromatic structure are not sp^2^ hybridized. Moreover, in the silk cocoon spectra, a wide peak is found at 20.4° corresponding to amorphous and crystalline protein β-sheet structure.^41^

This finding was further investigated by Raman spectroscopy. Graphene inherits highly delocalized π-electrons. Any modification to the spatial extent of the charge density of these π-electrons or tilt of the π-orbitals leads to significant changes to the physical properties of graphene.^42–45^ From the Raman spectra (Figure 3f), it is observed that the carbon structure of the pyrolyzed cocoon develops two broad carbon crystallite peaks manifesting around 1350 and 1580 cm^–1^, respectively. These two typical Raman bands of graphitic/graphene materials, namely, the D and G bands, respectively,^46,47^ appeared in the first-order region of the spectra.

The G band corresponds to C–C bond-stretching vibrations in the sp^2^ lattice graphite/graphene materials, whereas D is a defect-related band.^48^ The spectrum of pyrolyzed cocoon is like that of carbon soot.^49^

### 3.2 Characterization of P-GPy

SF films obtained from FA solution were investigated by FTIR analysis and indicated a secondary hydrated silk I structure with a crystalline structure that can be controlled by changing the pH of the solvent. After redissolving the SF films in PBS, the silk II structure is forming (Figure 4a,b). The broad peak observed at 3271 cm^–1^ and the peaks found at 1634 and 1548 cm^–1^ correspond to −OH stretching vibrations and C=O and N–H in-plane bending of amide I and amide II, respectively.^50^ The peaks observed at 1236 and at 1049 cm^–1^ are attributed to the random coil amide III and to the C–H bending of the protein molecule.^51^

**Figure 4:**
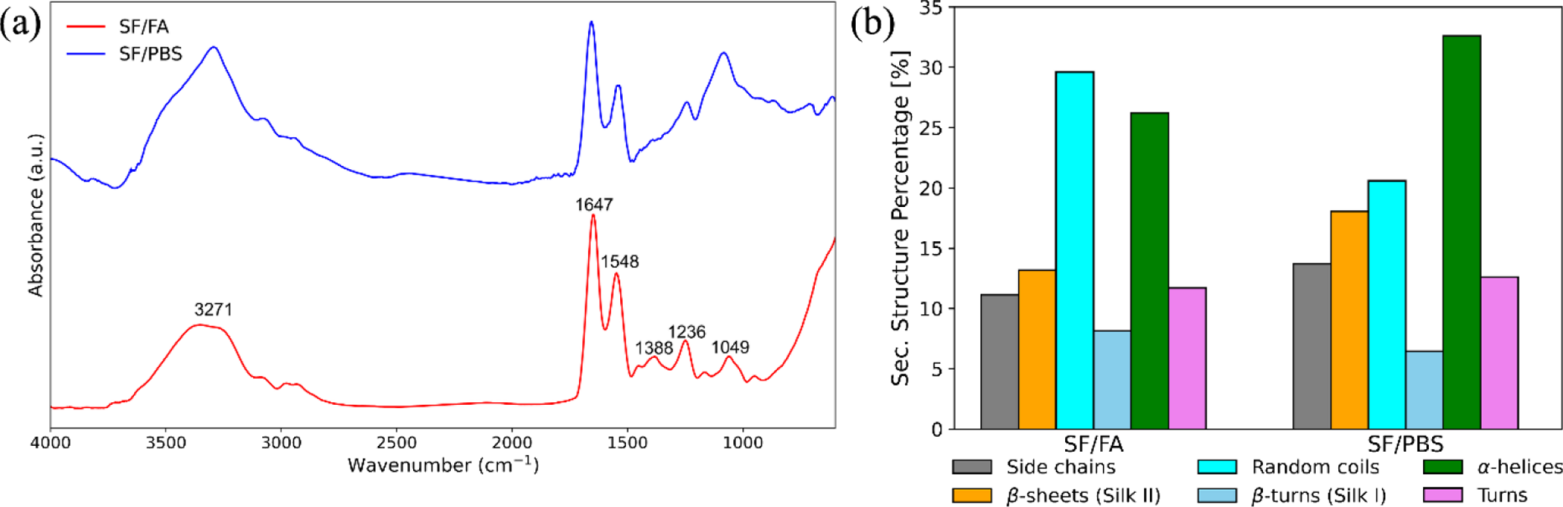
(a) ATR-FTIR spectra of SF films prepared in formic acid (SF/FA) and phosphate-buffered saline (SF/PBS). (b) Relative weight of components obtained by curve-fitting procedure of ATR-FTIR spectra of SF films prepared by different processes.

For the SF films prepared by ultrasound treatment, the total β-sheet content remains unaltered for the samples obtained from FA solution but increases for those redissolved in PBS. In these samples, the β-turn silk I structures decrease (Figure 4b), indicating the transition of an intermediate silk I structure to a silk II structure dominated by β-sheet antiparallel crystals.

Figure 5a and Figure 5b display, respectively, the SEM images and EDX results after the heat treatment of regenerated silk in PBS solution. As can be seen from Figure 5a, SEM images of the P-GPy show a cellular porous network with the phosphorus that is uniformly distributed across morphology (Figure 5c and Table 2).

**Figure 5:**
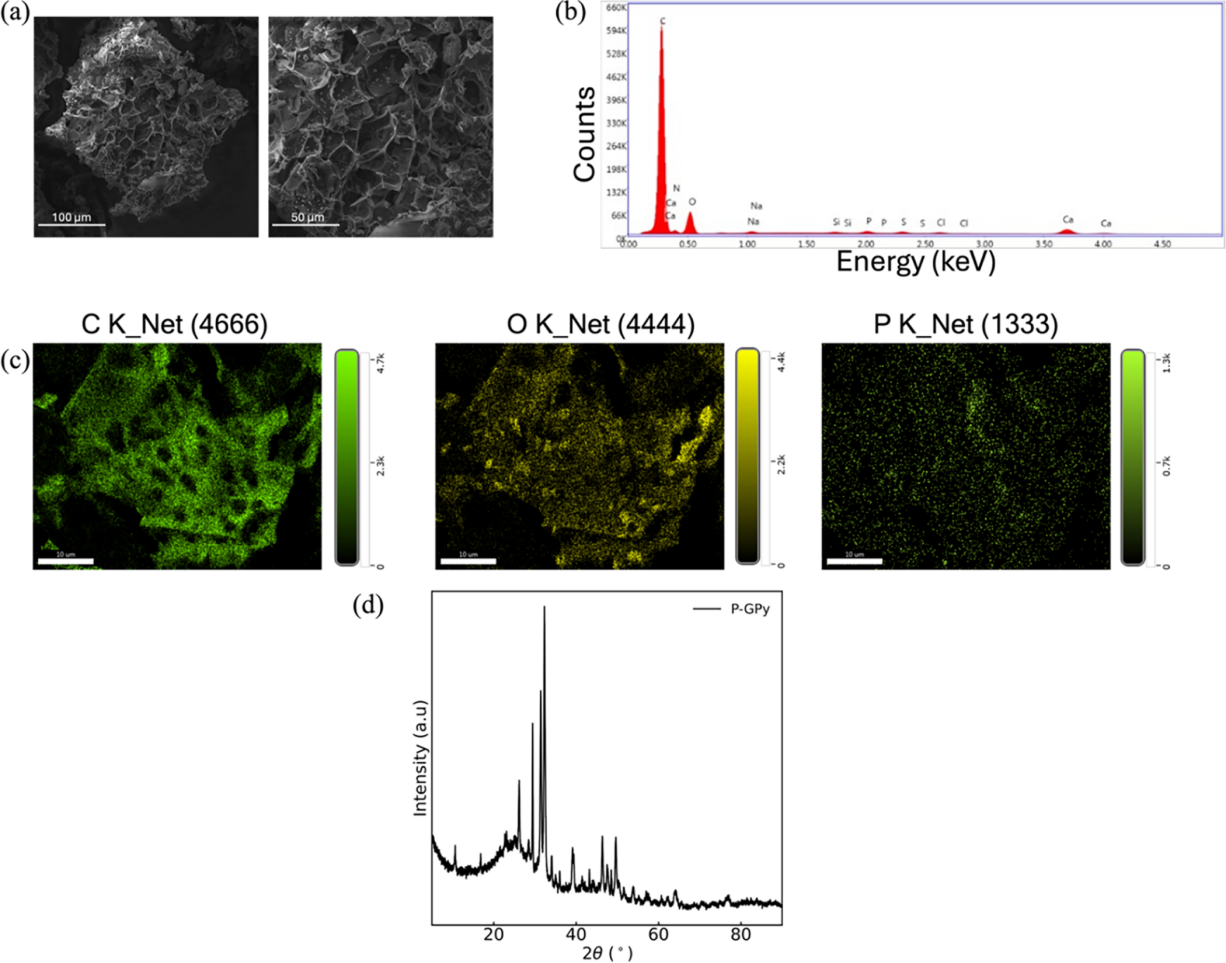
(a) SEM, (b) EDX spectrum, and (c) EDX mapping of bulk P-GPy after washing in water. (d) XRD pattern of bulk P-GPy.

**Table 2:** EDX Elemental Analysis of the P-GPy Sample

element	weight %	atomic %
C	78.37	84.62
N	1.67	1.54
O	14.51	11.76
Na	0.58	0.33
Si	0.28	0.13
P	0.67	0.28
S	0.56	0.23
Cl	0.42	0.15
Ca	2.94	0.95

XRD analysis conducted on the P-GPy after washing in water, as reported in Figure 5d, identifies the characteristic peaks of calcite (CaCO_3_) and chloroapatite (Ca_5_(PO_4_)_3_Cl) situated at 2θ = 24.2 and 27.3°, respectively, to the right of the pyrolyzed silk cocoon peak (23.6°).

### 3.3 Liquid Phase Exfoliation of P-GPy

Liquid phase exfoliation is a powerful method to produce exfoliated graphite in large scale using mild reaction conditions without complex post-treatments, as evidenced by recent studies.^52^ To study the morphology of the exfoliated P-GPy, SEM coupled with EDX analysis was carried out, as shown in Figure 6a,b and Table 3. The phosphate-intercalated pyroprotein possesses thick flakes in a stacked configuration (Figure 6a). Despite fluctuations in the intensity and location under varying experimental conditions, XRD peaks (Figure 6c) are indicative of intercalation products.

**Figure 6:**
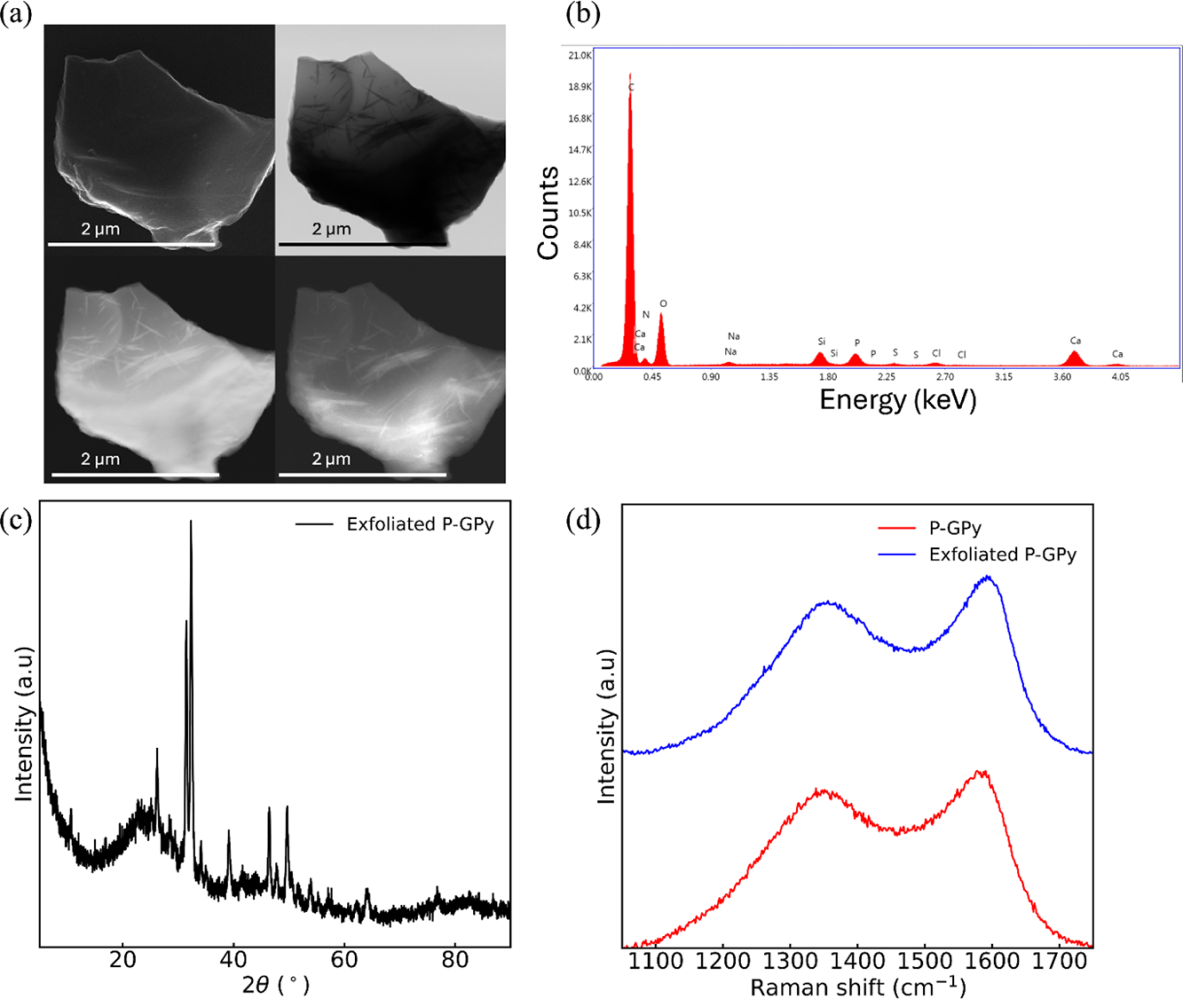
(a) SEM images, (b) EDX spectrum, and (c) XRD pattern of exfoliated P-GPy sample. (d) Comparison of Raman spectra of P-GPy and exfoliated P-GPy.

**Table 3:** Elemental Analysis of Exfoliated P-GPy

element	weight %	atomic %
C	66.27	75.13
N	4.55	4.42
O	19.64	16.72
Na	0.19	0.11
Si	1.48	0.72
P	1.84	0.81
S	0.25	0.11
Cl	0.51	0.2
Ca	5.28	1.79

The graphitic structure was evaluated by Raman spectroscopy, as shown in Figure 6d. Raman spectroscopy was used to assess changes in the G and D peak positions. In Figure 6d and Table 4, we show the Raman spectrum of intercalated P-GPy and deconvolution data, respectively. These results indicate that graphitic aromatic structures were formed from the β-sheet domains of SF. Raman spectra of the P-GPy and exfoliated P-GPy samples (Figure 6d and Figure S2) reveal a pseudographitic crystalline structure, with the integral intensity ratios of the D to G bands, that is, *I*_D1_/*I*_G_ (Table 4), passing from ∼3.1 for P-GPy to ∼2.21 for exfoliated P-GPy indicating the development of an sp^2^-hybridized carbon. Even if the temperature of the pyrolysis of SF is different from that adopted to fire the bricks, previous studies^5,6^ demonstrated that in the range between 600 and 900°, the graphitic structure of the pyroprotein is not influenced by the thermal treatment that starts to change above 1200 °C.

**Table 4:** Values of the Parameters Resulted from the Fitting Procedure of the Raman Spectra for the Three Types of Calcined Silk and the Value of the Ratio between the Intensities of the D1 and G Bands

		pyrolyzed cocoon	P-GPy	exfoliated P-GPy
G	position (cm^–1^)	1582	1591	1600
	width (cm^–1^)	42	32	33
D1	position (cm^–1^)	1331	1355	1369
	width (cm^–1^)	90	67	68
D2	position (cm^–1^)	1671	1663	1682
	width (cm^–1^)	23	12	20
D3	position (cm^–1^)	1510	1534	1539
	width (cm^–1^)	65	78	67
D4	position (cm^–1^)	1138	1252	1288
	width (cm^–1^)	89	84	87
*I*_D1_/*I*_G_		4.14	3.31	2.21

### 3.4 Piezo-Impedance and Piezoresistance Properties of P-GPy-Based Bricks

Figure 7 presents Nyquist plots of PCB and SFCB subjected to the minimum and maximum compressive loadings set for the electromechanical approach. Then, the curves for PCB present higher impedance than the SFCB curves, depicting an augment in electrical conductivity. In addition, SFCBs exhibit low diffusive components in the impedance spectra, all between copper tape electrodes and the porous structure, which accounts for the observed decrease in electrical resistance at low frequencies. This result confirms the formation of pyrolyzed SF, such that previous piezoresistive studies performed on SF/cement-based composites demonstrated a significant increase in electrical resistance.^35^

**Figure 7:**
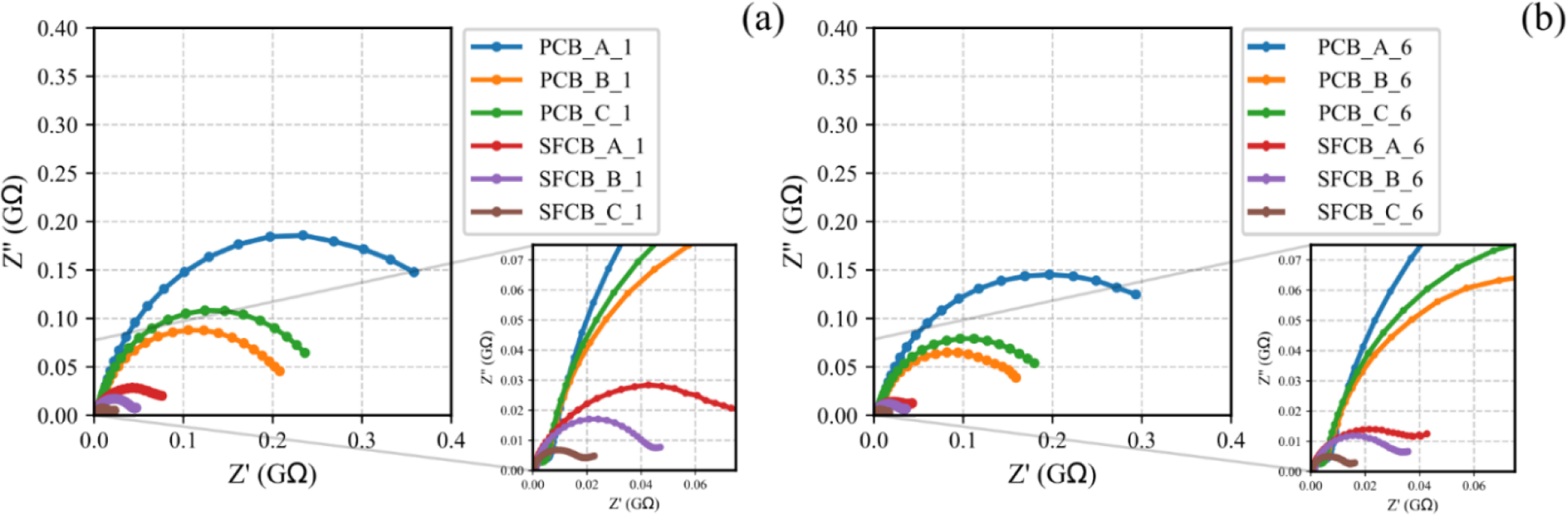
Nyquist plots of PCB (A, B, and C) and SFCB (A, B, and C) under compressive loading of (a) 0.025 and (b) 5 kN, labeled as 1 and 6 in the legends, respectively.

Nyquist semicircles of all smart bricks reduce their size under loading conditions, as depicted in Figure 7a,b. This is an indication of the decrease in electrical impedance over the frequency span. Consequently, the parameters in the circuit models are affected by force/strain variations.

After optimizing impedance data, electrical resistances R1, R2, and R3, and pseudoadmittances Q1, Q2, and Q3 were evaluated as a function of the force/strain (Figures S3 and S4). The best linearity was observed for parameters R2 and Q2, as shown in Figure 8. The electrical resistance R2 depicts the charge transfer resistance of the construction material,^53^ where the imaginary component of the impedance approaches zero. Furthermore, R2 of SFCB exhibited a better linearity (0.895) than PCB (0.273) in the least favorable scenario, and the charge transfer resistance decreased because of the addition of SF to clay-based bricks. Conversely, Q2 of PCB demonstrated a slightly better linearity and lower pseudoadmittance than SFCB.

**Figure 8:**
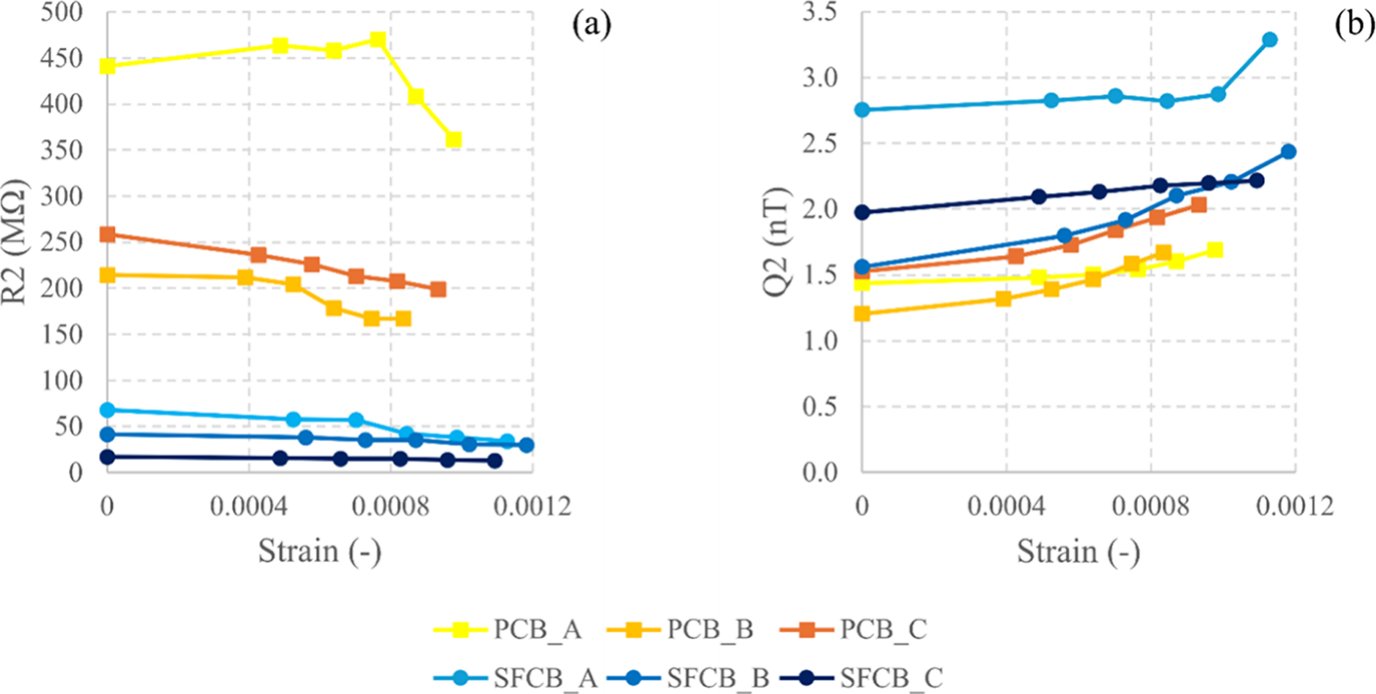
Strain sensitivity of (a) the midfrequency resistance (R2) and (b) pseudoadmittance (Q2). T units for pseudoadmittance can be expressed in terms of Ω^–1^ s^–*n*^, where *n* is the heterogeneity parameter of the constant phase element,^54^ ranging from 0 to 1.

On the other hand, electrical resistance of smart bricks responded with an analogous trend, showing a decrease in electrical resistance as the compressive force increased (see Figure 9a,b). However, in this alternative electrical characterization, the electrical resistance of SFCB is observed to be lower than that of PCB. Another important aspect is the presence of electrical noise accompanying the electrical resistance of PCB, suggesting that SFCB contains more conductive paths between the electrodes through the clay-based material. Previous studies of SF in cement-based composites revealed a type of memory effect during the unloading state, associated with the ion trapping.^35^ Nonetheless, SFCB did not exhibit this memory effect, likely due to the graphitization of SF structures during the high-temperature firing process.

**Figure 9:**
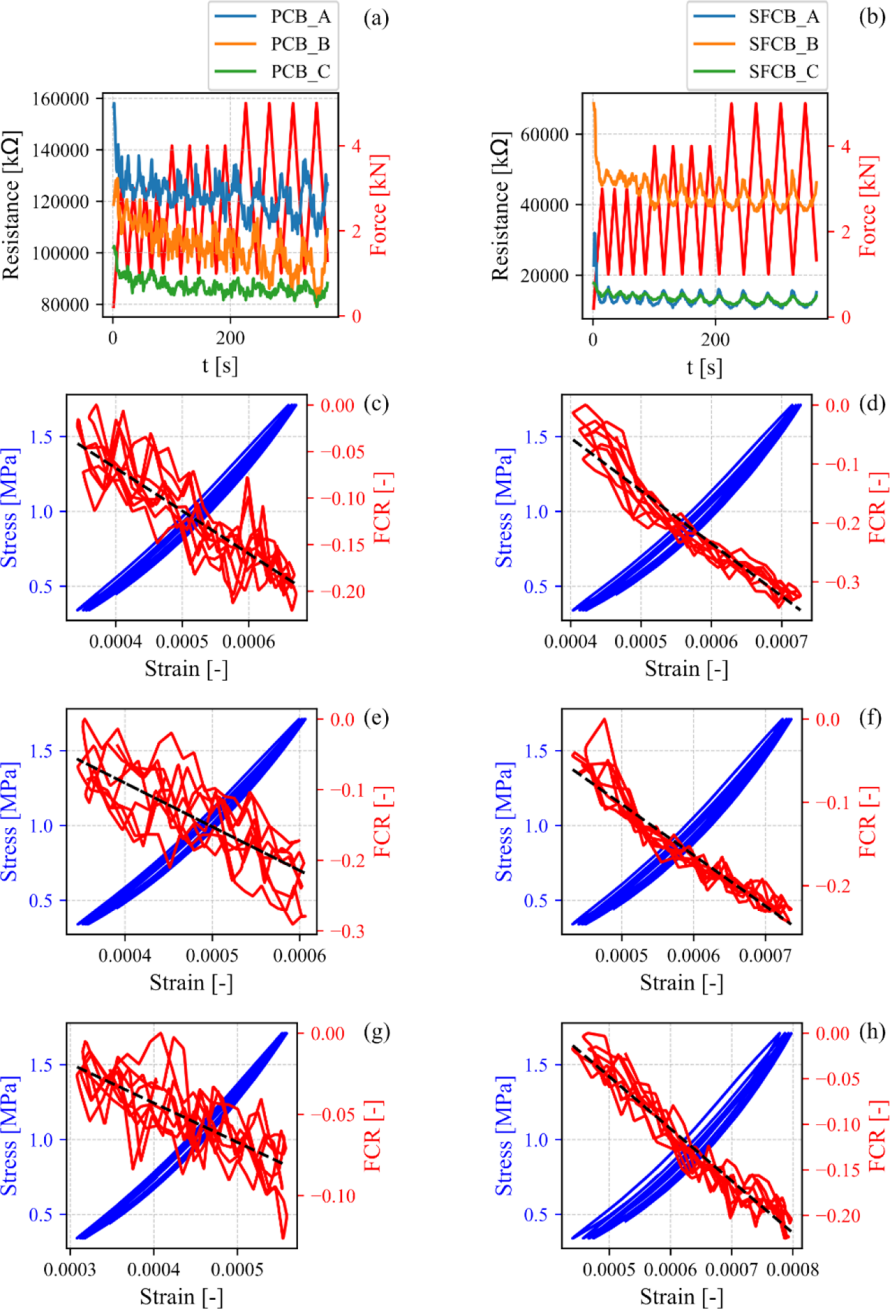
Piezoresistive response of (a) PCB and (b) SFCB over the time domain. Fractional change in electrical resistance and stress as a function of strain of (c) PCB_A, (d) SFCB_A, (e) PCB_B, (f) SFCB_B, (g) PCB_C, and (h) SFCB_C.

In Figure 9c–h shows the combined electromechanical response obtained by interpolating electrical and mechanical variables. The fractional change in electrical resistance (FCR) as a function of strain (ε) illustrates the strain sensitivity or gauge factor (λ) of the construction material. Accordingly, λ was calculated as follows:

$$\mathrm{FCR}=\frac{R_{0}-R\left(t\right)}{R_{0}}=\lambda \varepsilon$$ 3

Here, *R*_0_ denotes the electrical resistance under unloading conditions, while *R*(*t*) represents the electrical resistance during the loading state. Beyond repeatability and the magnitude of λ, linearity is a critical parameter in practical strain-sensing applications. In this context, the linearity of SFCB (represented by the coefficient of determination, *r* ^2^) was 0.960 ± 0.002, whereas PCB exhibited a lower linearity of 0.791 ± 0.053. Furthermore, the FCR increased by an average of 28% for SFCB followed by a 16% increase for PCB. This result denotes a 12% improvement in FCR due to the addition of SF to the clay-based material, combined with the ancient firing process involving carbon coke powder.

Figure 10 shows the gauge factors collected from the electromechanical methods proposed in this study: EIS-stair loading and biphasic cyclic loading. The biphasic approach (R_biph) revealed the highest gauge factor for SFCB, with a magnitude of 692.304 ± 143.546, compared to 440.432 ± 154.928 for PCB. However, in terms of reproducibility among equally prepared sensors, the charge transfer resistance R2 has demonstrated a better performance. In this case, the gauge factors for SFCB and PCB were approximately 311.503 ± 105.377 and 227.730 ± 73.148, respectively. On the other hand, the pseudo admittance Q2 led to a decrease in the gauge factor from 329.858 ± 128.636 for PCB to 230.855 ± 163.132 for SFCB.

**Figure 10:**
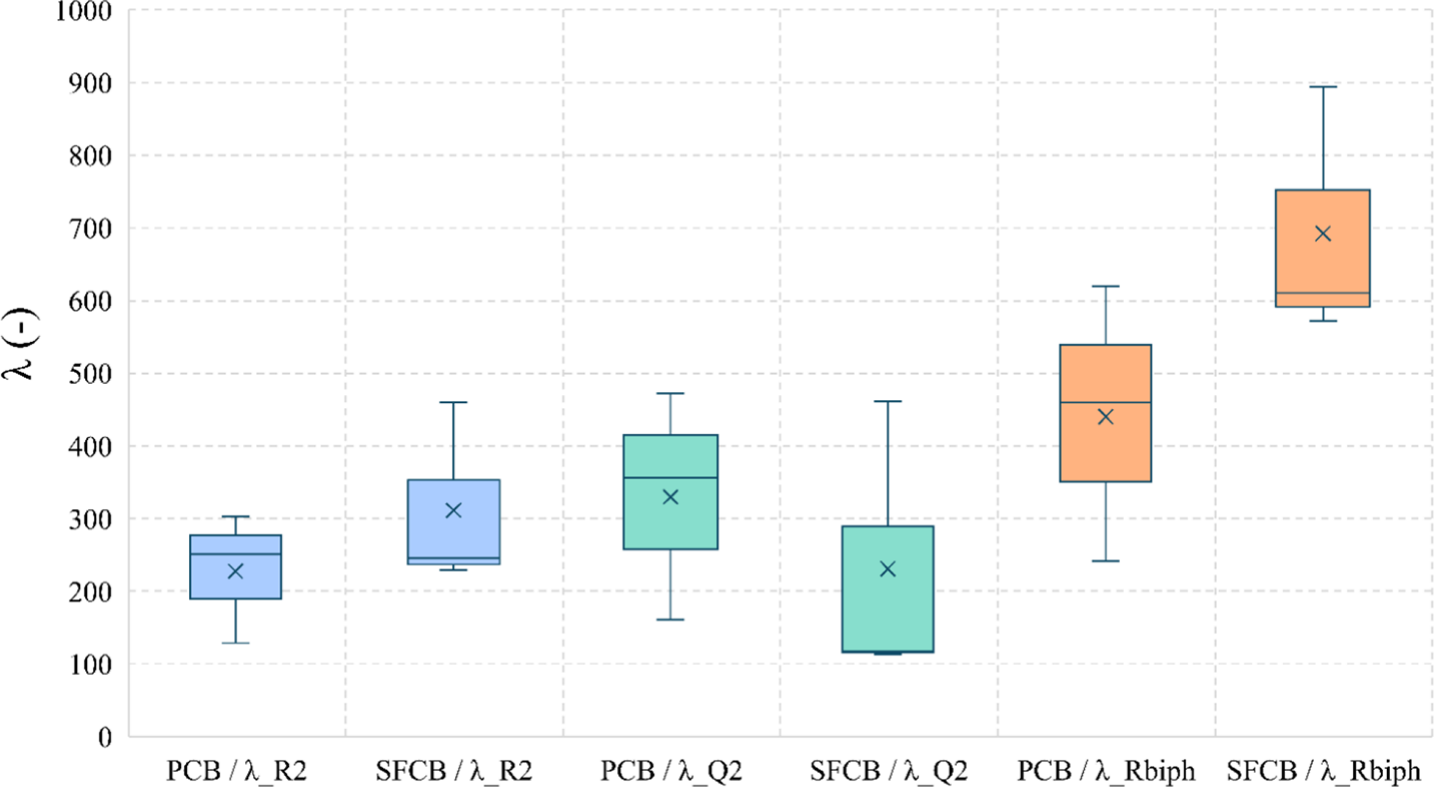
Gauge factors according to piezo-impedance parameters (R2), (Q2), and electrical resistance (*R*_biph_) obtained from the biphasic approach.

In terms of the average gauge factor across R2 and R_biph, there are significant increases of 37 and 57%, respectively. Prioritizing linearity and magnitude balanced with reasonable repeatability, SFCB, particularly R_biph, is the best parameter for strain-sensing applications. On the other hand, different configurations of smart bricks incorporating carbonaceous and metallic fibers have demonstrated gauge factors of the same order as SFCBs, as shown in Table 5. Nevertheless, SFCBs present similar benefits to CNF-based bricks in terms of fiber content, while titanium-based bricks exhibit a large difference in gauge factors between the same formulations. In fact, SFCBs exhibit good linearity, repeatability, and higher gauge factors per fiber weight than similar formulations reported in the literature.

**Table 5:** Gauge Factors for Silk Fibroin (SF), Titanium Carbon Nanofibers (CNF), Multiwalled Carbon Nanotubes (MWCNT), Graphene Nanoplatelets (GNP), Titanium Powder, and Steel Fiber-Doped Bricks

fiber	wt. %	λ
SF (this study)	0.1	692
CNF^55^	0.2	956
MWCNT^55^	0.2	216
GNP^55^	0.2	122
titanium powder^56^	5	391 to 2955
steel fibers^57^	0.25	374

## 4 Conclusions

In summary, we demonstrated that the intrinsic β-sheet structures of silk proteins can be transformed to graphitic structures. Exploiting the regeneration process of silk fibers, we show that large molecules, such as calcium phosphate, can intercalate the layered graphitic pyroprotein planes, forming a heterointerface. This was evident from the strong Ca, P, and O signals that were detected in pyrolyzed silk fibroin from spectroscopic measurements (EDX and XRD). Moreover, the intercalated phosphate pyroprotein can also be exfoliated with a sustainable water process. Additionally, pyroproteins were combined with clay-based composites to fabricate smart bricks with piezoresistive capabilities. Electromechanical characterizations highlight the potential of this approach for strain-sensing capabilities improving the gauge factor by 20% in comparison to the reference composites, which can be further integrated into future structural health monitoring applications. The concept of piezo-impedance was also introduced in this work, expanding the set of strain-sensitive electrical parameters. Nevertheless, the biphasic approach exhibited approximately twice the sensitivity of pseudo admittance at midfrequency range, as indicated by the gauge factors.

## Supplementary Material



## Notes

(Figure S1) (a) Scanning electron microscopy of silk cocoon and (b) SF films produced by leaving the SF solutions to evaporate onto Petri dishes for 48 h; (Figure S2) deconvoluted Raman spectra of P-GPy exfoliated in (a) DMSO and (b) water excited at 633 nm; (Table SI) values of the parameters resulted from the fitting procedure of the Raman spectra for P-GPy exfoliated in water, and the value of the ratio between the intensities of the D1 and G bands; (Figure S3) Nyquist diagrams of SF/clay-based composites subjected to different force levels; and (Figure S4) strain sensitivity of electrical parameters extracted from the circuit models shown in Figure 2a,b (PDF)
