# Supplementary material for: Water-Processable Intercalated Graphitic Pyroproteins and Electromechanic Properties for Smart Structural Bricks
Source: ACS Omega. 2025 Oct 27;10(44):53212–23. doi: 10.1021/acsomega.5c07891 (PMC12613132; doi:10.1021/acsomega.5c07891)
Supplement: Supplementary file 1 [file ao5c07891_si_001.pdf]

Supporting Information

Water processable intercalated graphitic pyroproteins and electromechanics properties for smart structural bricks ”

Maria Caporali[a], Daniel A. Triana-Camacho[b], Rocco Malaspina[c], Antonella D’Alessandro[b], Andrea Meoni[b], Andrea Ienco[a], Stefano Martinuzzi[a], Martina Banchelli[d], Filippo Ubertini[b], Luca Valentini[b]*

[a] Institute of Chemistry of OrganoMetallic Compounds-ICCOM, National Research Council-CNR, 50019 Sesto Fiorentino, Italy

[b] Department of Civil and Environmental Engineering, University of Perugia, Via G. Duranti, 06125 Perugia, Italy

[c] Department of Physics and Geology, University of Perugia, Via A. Pascoli, 06123 Perugia, Italy

[d] IFAC, Istituto di Fisica Applicata Nello Carrara, Via Madonna del Piano 10, Sesto Fiorentino, Italy

*E-mail: luca.valentini@unipg.it

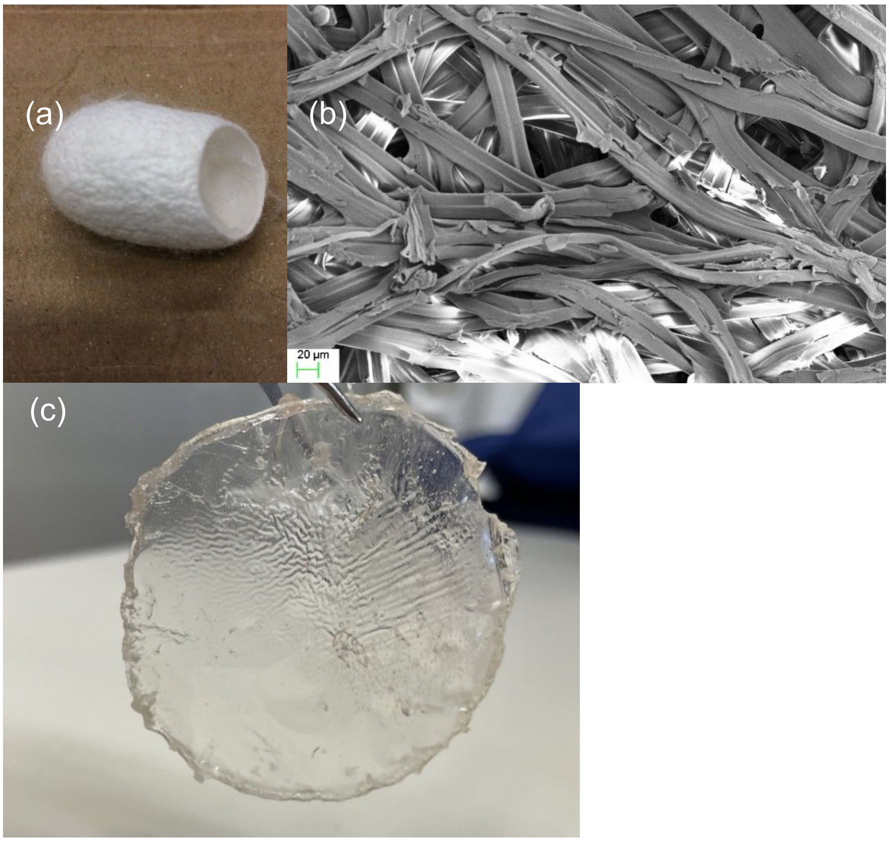

Figure S1. (a) Silk cocoon, (b) scanning electron microscopy of silk cocoon and (c) SF film produced by leaving the SF solution to evaporate onto Petri dishes for 48 hours.

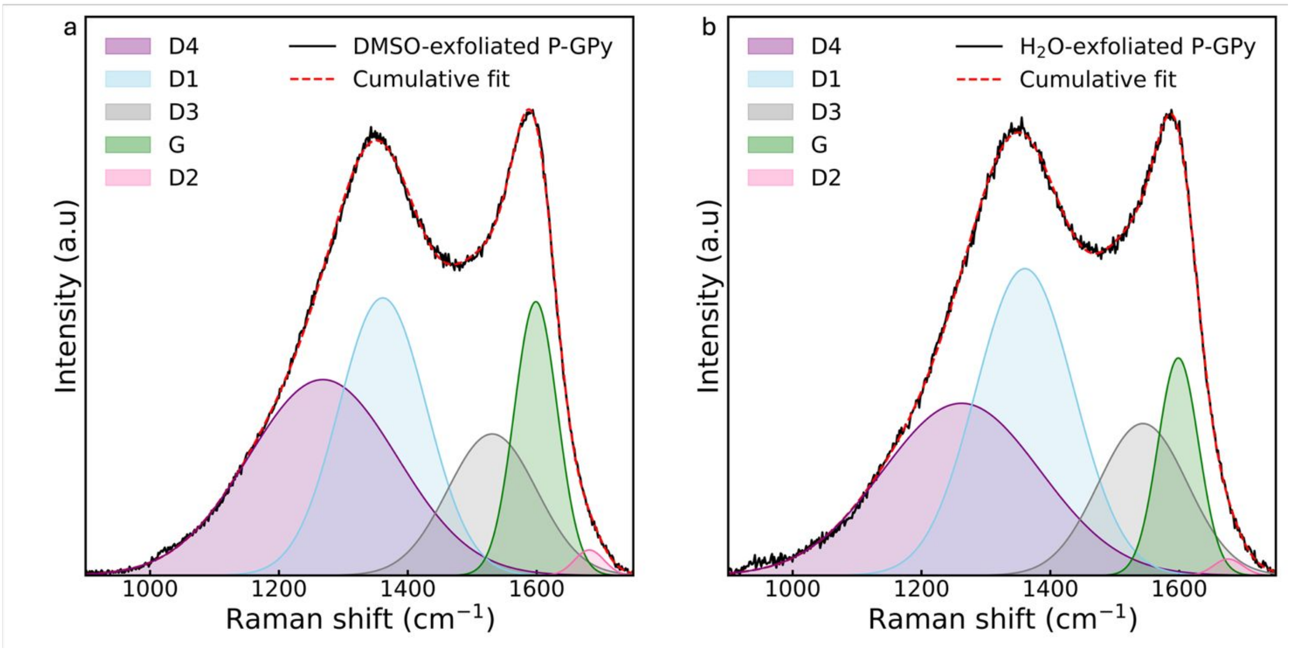

Figure S2. Deconvoluted Raman spectra of P-GPy exfoliated in (a) DMSO and (b) water excited at 633 nm.

Table SI. Values of the parameters resulted from the fitting procedure of the Raman spectra for P-GPy exfoliated in water, and the value of the ratio between the intensities of the D1 and G bands.

		DMSO-exfoliated P-GPy	H_2O-exfoliated P-GPy
**G**	Position (cm^{-1})	1599	1599
	Width (cm^{-1})	33	32
**D1**	Position (cm^{-1})	1361	1361
	Width (cm^{-1})	68	76
**D2**	Position (cm^{-1})	1682	1676
	Width (cm^{-1})	23	23
**D3**	Position (cm^{-1})	1531	1543
	Width (cm^{-1})	69	69
**D4**	Position (cm^{-1})	1268	1262
	Width (cm^{-1})	115	122
I_{D1}/I_G		2.06	3.40

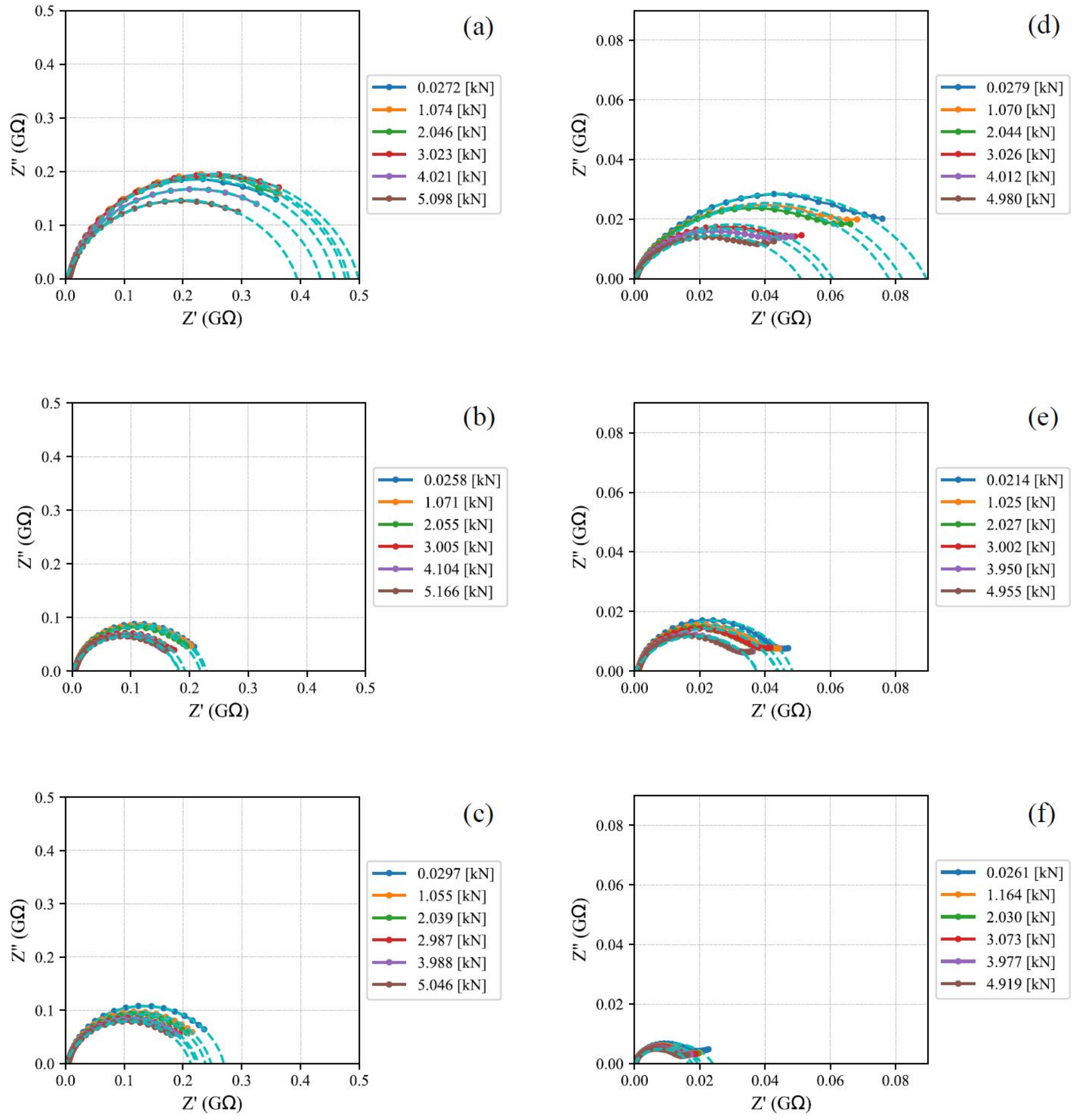

Figure S3. Nyquist diagrams of SF/clay-based composites subjected to different force levels.

Specimens: (a) PCB_A, (b) PCB_B, (c) PCB_C, (d) SFCB_A, (e) SFCB_B, and (f) SFCB_C.

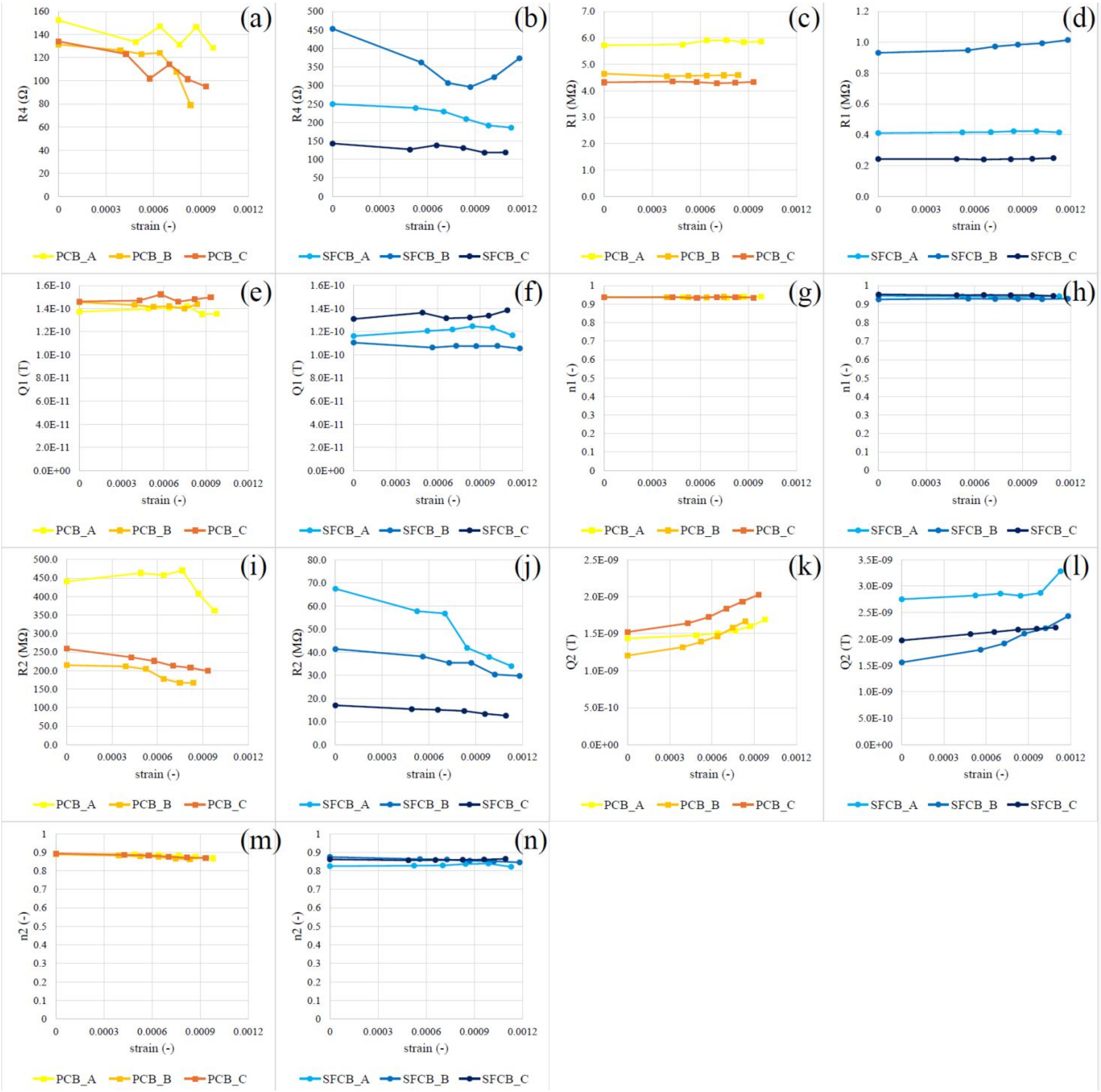

Figure S4. Strain sensitivity of electrical parameters extracted from the circuit models shown in Figures 2a and 2b. The complete set of parameters includes: (a), (b) high-frequency resistance (R4); (c), (d) Cole-Cole resistance (R1); (e), (f) high-frequency pseudo-admittance (Q1); (g), (h) high-frequency heterogeneity factor (n1); (i), (j) mid-frequency resistance (R2); (k), (l) mid-frequency pseudo-admittance (Q2); and (m), (n) mid-frequency heterogeneity factor (n2).
